# The ClinGen Severe Combined Immunodeficiency Disease Variant Curation Expert Panel: Specifications for classification of variants in *ADA*, *DCLRE1C*, *IL2RG*, *IL7R*, *JAK3*, *RAG1*, and *RAG2*

**DOI:** 10.1016/j.gim.2025.101613

**Published:** 2025-10-14

**Authors:** Vanessa C. Jacovas, Michelle Zelnick, Shannon McNulty, Justyne E. Ross, Namrata Khurana, Xueyang Pan, Alejandro Nieto, Shiloh Martin, Benjamin McLean, Marwa A. Elnagheeb, Morton J. Cowan, Jennifer M. Puck, Mike S. Hershfield, James Verbsky, Jolan Walter, Eric J. Allenspach, Alice Y. Chan, Nicolai S.C. van Oers, Rajarshi Ghosh, Megan Piazza, Bo Yuan, Luigi D. Notarangelo, Britt A. Johnson, Ivan K. Chinn, Severe Combined Immunodeficiency Variant Curation Expert Panel

**Affiliations:** 1 Baylor College of Medicine, Houston, TX;; 2 Division of Pediatric Immunology, Allergy, and Retrovirology, Texas Children’s Hospital, Houston, TX;; 3 Department of Genetics, University of North Carolina, Chapel Hill, NC;; 4 Invitae Corporation, San Francisco, CA;; 5 Department of Pediatrics, Division of Pediatric Allergy, Immunology and Bone Marrow Transplantation, University of California San Francisco Benioff Children’s Hospital, San Francisco, CA;; 6 Department of Medicine and Biochemistry, Duke University School of Medicine, Durham, NC;; 7 Division of Rheumatology, Department of Pediatrics, Medical College of Wisconsin and Children’s Wisconsin, Milwaukee, WI;; 8 Division of Pediatric Allergy/Immunology, University of South Florida at Johns Hopkins All Children’s Hospital, St. Petersburg, FL;; 9 Division of Immunology, Department of Pediatrics, University of Washington and Seattle Children’s Research Institute, Seattle, WA;; 10 Department of Immunology, Pediatrics and Microbiology, University of Texas Southwestern Medical Center, Dallas, TX;; 11 Division of Intramural Research, National Institute of Allergy and Infectious Diseases, National Institutes of Health, Bethesda, MD;; 12 PreventionGenetics, Part of Exact Sciences, Marshfield, WI;; 13 Human Genome Sequencing Center, Baylor College of Medicine, Houston, TX

**Keywords:** ACMG/AMP guidelines, ClinGen, SCID

## Abstract

**Purpose:**

This collaborative study, led by the Clinical Genome Resource Severe Combined Immunodeficiency Disease Variant Curation Expert Panel (ClinGen SCID-VCEP), implemented and adapted the American College of Medical Genetics and Genomics/Association for Molecular Pathology (ACMG/AMP) guidelines for interpreting germline variants in genes with established relationships to SCID. The effort focused on the 7 most common SCID-related genes identified by SCID newborn screening in North America: *ADA*, *DCLRE1C*, *IL2RG*, *IL7R*, *JAK3*, *RAG1*, and *RAG2*.

**Methods:**

The SCID-VCEP conducted a rigorous review of variants that involved database analyses, literature review, and expert feedback to derive gene-specific modifications to the ACMG/AMP guidelines. These specifications were validated using a pilot set of 90 variants.

**Results:**

Of these 90 variants, 25 were classified as pathogenic, 21 as likely pathogenic, 14 as variants of uncertain significance, 18 as likely benign, and 12 as benign. Seventeen variants with conflicting classifications in ClinVar were successfully resolved. The criteria included modifications to 20 of the 28 original ACMG/AMP criteria specific to SCID-related genes.

**Conclusion:**

The SCID-specific variant curation guidelines developed by the SCID-VCEP will enhance the precision of SCID genetic diagnosis and provide a robust framework for interpreting variants in SCID-related genes, contributing to appropriate treatment of SCID.

## Introduction

Severe Combined Immunodeficiency Disease (SCID) represents a major clinical challenge due to severely compromised immunity that makes newborns extremely vulnerable to life-threatening infections.^1^ Affecting approximately 1 in every 60,000 births in North America,^2^ SCID requires early diagnosis and intervention for survival. Without timely and appropriate treatment, SCID is usually fatal within the first year of life. However, the advent of population-based newborn screening now identifies SCID soon after birth, significantly improving the prospects for successful treatment.^3 ,4^ The 2022 International Union of Immunologic Societies (IUIS) classification of inborn errors of immunity summarized the genetic diversity of SCID, linking it to variants in 19 different genes and underscoring the complexity inherent to diagnosing and treating this condition.^5^

The treatment strategies for SCID are intricately linked to the genetic pathways affected, varying from enzyme replacement therapy to hematopoietic stem cell transplantation or gene therapy.^6^ This genetic complexity necessitates genetic testing and precise interpretation for the development of optimal treatment plans. The introduction of advanced gene sequencing technologies has increased the challenge of classifying gene variants by revealing a higher number of rare and novel variants that require further analysis and validation, thus making accurate curation a cornerstone in avoiding diagnostic errors and ensuring the deployment of suitable treatment pathways.

In response to this challenge, ClinGen^7^ has devised a framework to establish EPs tasked with refining the 2015 American College of Medical Genetics and Genomics/Association for Molecular Pathology (ACMG/AMP) sequence variant interpretation guidelines for specific gene-disease relationships.^8^ Recognizing the pivotal role of accurate genetic diagnosis in SCID, the ClinGen SCID Variant Curation Expert Panel (SCID-VCEP) was established in 2019. This expert panel is dedicated to adapting the ACMG/AMP guidelines for variant classification in the context of SCID, focusing first on 7 genes that are most frequently implicated in SCID patients in North America and accounting for 80% of all reported cases captured by the Primary Immune Deficiency Treatment Consortium (PIDTC)^9^ : *ADA* (adenosine deaminase, HGNC:186), *DCLRE1C* (DNA cross-link repair 1C, HGNC:17642), *IL2RG* (interleukin 2 receptor gamma subunit, HGNC:6010), *IL7R* (interleukin 7 receptor, HGNC:6024), *JAK3* (Janus kinase 3, HGNC:6193), *RAG1* (recombination activating gene 1, HGNC:9831), and *RAG2* (recombination activating gene 2, HGNC:9832).^10^

This article presents the formulation and refinement of variant classification processes to develop an improved curation framework for these critical genes. By submitting variant classifications to ClinVar as an “expert panel” submitter, resolving discrepancies, moving variants of uncertain significance (VUS) toward benign or pathogenic status, and enhancing confidence in SCID variant classifications in ClinVar, this work marks a significant step forward in the genetic diagnosis and treatment of SCID, ultimately helping to improve patient care and management in this challenging field.

## Material and Methods

### Creation of the SCID-VCEP

The SCID-VCEP^11^ was established in November 2019 and is affiliated with the ClinGen Immunology Clinical Domain Working Group. The SCID-VCEP was assembled to include multidisciplinary experts who have expertise in inborn errors of immunity: immunologists, clinical and molecular geneticists, genetic counselors, research scientists, and variant curation experts. All EP members disclosed potential conflicts of interest as required. The VCEP scope of work was designated as genes with established relationships to a SCID phenotype, especially as listed in Table 1 of the IUIS document,^5^ focusing initially on 7 genes: *ADA*, *DCLRE1C*, *IL7R*, *JAK3*, *RAG1*, *RAG2* (all autosomal recessive mode of inheritance, AR), and *IL2RG* (X-linked recessive mode of inheritance, XLR).

### ACMG/AMP specifications

Adapting the ACMG/AMP sequence variant interpretation guidelines^8^ for the 7 target genes in relation to SCID was systematically developed through monthly teleconferences. The SCID Variant Curation Expert Panel (VCEP) meticulously reviewed each criterion in the ACMG/AMP guidelines, leading to the establishment of 7 sets of rules or specifications, 1 for each gene. The finalized specifications received endorsement from the Sequence Variant Interpretation (SVI) Working Group and are publicly accessible on the Criteria Specification Registry (CSpec).^12^ The ACMG/AMP specifications are updated periodically; therefore, to find the most current information please visit https://cspec.genome.network.

### Validation and pilot testing

The specifications for the 7 genes were validated using 90 pilot variants distributed nearly equally across the 7 genes. Variants were nominated by SCID-VCEP members from ClinVar and internal laboratory data, with the objective of including a balance of known/suspected pathogenic or benign variants (75.6%) and VUS or variants with conflicting/missing assertions (24.4%). Additionally, variants were selected to represent various variant types: missense (52.2%), nonsense and frameshift (21.1%), synonymous (13.4%), splicing (8.9%), and intronic (4.4%). All pilot variants were annotated using RefSeq IDs NM_000022.4 (*ADA*), NM_001033855.3 (*DCLRE1C*), NM_000206.3 (*IL2RG*), NM_002185.5 (*IL7R*), NM_000215.4 (*JAK3*), NM_000448.3 (*RAG1*), and NM_000536.4 (*RAG2*).

Two trained biocurators independently evaluated each variant according to the SCID-VCEP standards using the ClinGen Variant Curation Interface^13^ to record criteria and evidence. Three American Board of Medical Genetics and Genomics (or foreign equivalent) certified geneticists and one immunologist expert in the gene of interest independently reviewed this evidence, with final decisions made by consensus. Following step 4 approval from ClinGen, these variants were then deposited into ClinVar with a 3-star rating, indicating expert panel review.

## Results

This article presents the results of the SCID-VCEP refinement process for adapting the ACMG/AMP variant interpretation guidelines to analyze variants genes with established relationships to SCID. The resulting specifications for the classification of germline variants in *ADA*, *DCLRE1C*, *IL2RG*, *IL7R*, *JAK3*, *RAG1*, and *RAG2* were approved by the SVI Working Group (July 20, 2023) and serve as a framework to guide the assessment of variant pathogenicity in these genes.

Of the 28 original ACMG/AMP criteria, 8 (BP1, BP2, BP3, BP4, BP5, BP6, PP2, and PP5) were determined not applicable for these genes and were excluded from the framework. These criteria were excluded because of gene-specific features (eg, frequent pathogenic missense variation, lack of validated in silico tools, and absence of repetitive sequences) and adherence to ClinGen recommendations regarding inheritance patterns and external classifications. Another 20 of the 28 original criteria required gene and/or disease-specific alterations (BA1, BP7, BS1, BS2, BS3, PM1, PM2, PM3, PM4, PM5, PM6, PP1, PP3, PP4, PS1, PS2, PS3, PS4, and PVS1). Only 1 criterion was approved without additional modifications (BS4). This section presents a comprehensive presentation of the criteria along with specific details.

### Modification of the ACMG/AMP criteria based on specific classes of criteria

#### Variant frequency and use of control populations (BA1, BS1, BS2, PM2, and PS4)

SCID is a rare condition with variable prevalence across North America, ranging from 1 in 58,000 newborns (95% CI: 1 in 46,000–80,000)^14^ to 1 in 65,000 (95% CI: 1 in 51,000–90,000).^15^ However, its incidence markedly increases in certain ethnic groups, particularly in the Middle East where high rates of consanguinity and limited genetic diversity—features of a pronounced population structure—lead to a significantly higher prevalence, reaching 1 in 5000 live births.^14,16^

To establish stringent requirements for BA1 (allele frequency is greater than expected for disorder, Stand-alone), we used the highest reported prevalence of 1 in 5000 in the Middle Eastern population.^16^ For BS1 (allele frequency is greater than expected for disorder), we utilized a general number for the North American population and defined it as 1 in 50,000.^14 ,15^ The penetrance, prevalence, and allelic/genetic heterogeneity used for each gene are displayed in Table 1 and were calculated using the CardioDB metrics allele frequency web tool.^17^

The SCID-VCEP recommends using the Genome Aggregation Database (gnomAD) Grpmax Filtering Allele Frequency to assess BA1, BS1, and PM2; if unavailable, it should be based on the highest minor allele frequency considering all nonbottleneck populations. Given the absence of distinctly higher SCID incidence in any gnomAD bottleneck populations, for example, Ashkenazi Jewish and Finnish, we enabled the assessment of allele frequency in these populations for BS1.

Within our framework, the PM2 criterion (absent from population databases), which provides evidence for variant pathogenicity based on absent or extremely rare minor allele frequency, was downgraded to supporting strength per SVI Working Group guidelines.^18^ For *ADA*, *DCLRE1C*, *IL7R*, and *JAK3*, the thresholds were based on the most frequent pathogenic variant present in gnomAD, excluding known founder variants. The pathogenic classifications were derived using other SCID-VCEP specifications while excluding PM2 as a criterion to avoid circularity. Regarding *RAG1* and *RAG2*, which both comprise a single exon, achieving the pathogenic classification in absence of PM2 proved challenging without applying PVS1 (predicted null variant in a gene in which loss of function is a known mechanism of disease) at its maximum strength. Furthermore, for *IL2RG*, only 1 likely pathogenic variant was listed in gnomAD, and it did not reach the pathogenic level using SCID-VCEP specifications. Therefore, for these 3 genes, *RAG1*, *RAG2*, and *IL2RG*, we applied a second approach based on CardioDB calculations by reducing the allelic heterogeneity parameter. Table 1 contains all thresholds and settings. Additionally, to apply PM2, the variant cannot be observed in the homozygous state within any population.

Criterion PS4 (prevalence of the variant in affected individuals is significantly increased compared with the prevalence in controls) is based on the significantly higher prevalence of a variant in case cohorts versus control cohorts, which is considered strong evidence for pathogenicity. Ideally, published case-control studies are used as evidence. Most pathogenic or likely pathogenic (P/LP) variants in these genes are rare, and because limited case-control data are available for most variants, the VCEP recommends counting individual cases toward PS4. This PS4 criterion is specifically applied to *IL2RG*, which is XLR. Because the other 6 genes are AR, the PM3 criterion is utilized to count probands (see PM3 section below). To apply the PS4 code for *IL2RG*, the proband must meet SCID diagnostic criteria according to the PIDTC definitions for SCID^1^ (Supplemental Table 1), and the variant must be sufficiently rare (meet PM2 criteria). The *IL2RG* PS4 specification has a sliding weight scale to account for the number of unrelated probands who meet the SCID diagnostic criteria. PS4 is applied with Very Strong strength when described in ≥4 unrelated probands, default Strong strength with 3 probands, Moderate strength with 2 probands, and Supporting strength with 1 proband. To avoid double counting of probands, the proband evaluated for PP4 is excluded from PS4 counting.

The BS2 criterion (observed in a healthy adult individual with full penetrance expected at an early age) assesses the presence of a particular variant in a healthy adult, in which full penetrance of the associated condition is expected at an early age. Identifying a variant in the homozygous state within a healthy adult provides strong evidence supporting a benign interpretation. Given that SCID is often diagnosed at birth through newborn screening programs, we tailored the application of this criterion to align with the inheritance pattern of the gene. For AR genes (*ADA*, *DCLRE1C*, *IL7R*, *JAK3*, *RAG1*, and *RAG2*), the BS2 criterion is applied at a supporting level when at least 1 unaffected adult individual is documented in the literature or population databases, such as gnomAD, carrying the variant in the homozygous state. For the *IL2RG* gene, which is XLR, to apply BS2 at the supporting level, the variant must be observed in at least 2 unaffected hemizygous individuals. BS2 may be applied at the strong level if a variant is observed in 3 or more unaffected individuals.

#### Functional data/experimental (BS3 and PS3)

For PS3 (well-established in vitro or in vivo functional studies support a damaging effect on the gene or gene product) and BS3 (well-established in vitro or in vivo functional studies show no damaging effect on protein function or splicing), we adopted the system previously described^19^ to set specific minimum quality standards for in vitro functional assays.

The VCEP first conducted literature reviews and identified the most prevalent categories of functional assays used for these genes: enzyme activity, recombination and repair activity, phosphorylation and binding assays, surface expression, and interaction profiling (Supplemental References 1–19). For each assay, a review of the literature was used to define quality criteria by which the strength of the evidence provided could be assessed, including the number of basic controls, technical replicates, positive controls, and negative controls (Supplemental Material 1).

The BS3 criterion is exclusively applied to *ADA*, based on the high specificity of enzymatic measurement of ADA activity. Conversely, for *IL2RG*, *IL7R*, *JAK3*, *DCLRE1C*, *RAG1*, and *RAG2* only PS3 is applicable. Additionally, the PS3 criterion may be applied at a default strength level of “strong” when supported by data from an animal model that express the variant in question and recapitulate the SCID phenotype. Table 2 provides a summary of the assays utilized and the requisite strength of evidence for each gene, as determined by these criteria.

#### Critical domain (PM1)

PM1 (mutational hot spots and well-established functional domains) is defined by the SCID-VCEP for use at varying strength levels, from supporting to strong, depending on the gene and the region affected. Well-established functional domains were determined based on the known functions of the proteins and basic research defining these domains. For *ADA*, *DCLRE1C*, and *IL7R*, PM1 is not applicable because of the absence of such regions in these genes. For *IL2RG*, *JAK3*, *RAG1*, and *RAG2*, the relevant regions and the strength of their applicability are detailed in Table 3.^20–22^

#### Computational and predictive data (PVS1, PM4, PP3, BP7, PM5, and PS1)

The PVS1 criterion (predicted null variant in a gene in which loss of function is a known mechanism of disease) evaluates the impact of loss-of-function variants on gene function and potential pathogenicity. The level of evidence it provides toward pathogenicity can range from “very strong” to “supporting,” depending on where in the gene the variant occurs. The ClinGen SVI Working Group has provided detailed guidelines for how to apply this criterion effectively.^23^ In accordance with the published PVS1 flowchart, we specified PVS1 on the basis of well-established evidence for our 7 genes, based on their specific characteristics and genomic locations. The flowcharts depicting all types of loss-of-function variants and specific gene adaptations can be found in Supplemental Material 2.

Additionally, the PM4 criterion is applicable to in-frame deletions or insertions smaller than an entire exon, as well as in-frame whole-exon duplications that do not meet the PVS1 criteria. For PM4 to be applied at its default strength to deletion variants, the affected region must encompass a variant that has been previously established as pathogenic or likely pathogenic without predictions or observations of altered splicing. Conversely, if the region contains a VUS that is also not predicted or observed to affect splicing, then PM4 may be applied at a supporting level, denoted as PM4_Supporting.

The SCID-VCEP decided not to utilize in silico predictors for the PP3 criterion when evaluating missense variants due to insufficient validated evidence regarding the efficacy of these tools in this specific gene set. The PP3 criterion may still be applied for synonymous or intronic variants that are predicted by SpliceAI to affect splicing, provided that they exhibit a delta score greater than or equal to 0.2.^24^

The group agreed to adopt BP7, as delineated by ACMG/AMP, for synonymous variants in which splicing prediction algorithms anticipate no effect and to broaden the criterion to encompass intronic variants situated at or beyond the +7 or −21 nucleotide positions. For a variant to meet BP7, it must be forecasted by a minimum of 2 out of 3 computational tools to have no influence on splicing. Considering the possible low conservation of nucleotides across vertebrate genes associated with T cell and B cell development, the presence of nucleotide conservation is not a prerequisite for the application of BP7.

In the context of variants affecting the same amino acid (AA) residue, the SCID-VCEP refined the application of the ACMG/AMP criteria by introducing nuanced recommendations for 2 rules: PS1 and PM5. For PS1 (same AA change as a previously established pathogenic variant), a new strength level of PS1_moderate was introduced for changes corresponding to a variant deemed likely pathogenic. Conversely, the PM5 criterion is applied with supporting strength (PM5_Supporting) when a different missense change at the same residue has been previously classified as likely pathogenic. The previously established variant must have been designated as pathogenic or likely pathogenic using SCID-VCEP specific standards.

#### Case/Segregation

##### Segregation data (PP1 and BS4)

PP1 (cosegregation with disease in multiple affected family members) is applied according to the guidelines developed by the ClinGen Hearing Loss VCEP in collaboration with the SVI. This approach considers the logarithm of the odds (LOD) score and the number of affected and unaffected segregations to determine whether PP1 can be used at a supporting, moderate, or strong level.^25^ A LOD score of ≥0.6 to <1.2 is required to use PP1 at a supporting level, a score of ≥1.2 to <1.5 for a moderate level, and a score of ≥1.5 for a strong level.

BS4 (lack of segregation in affected members of a family) can be applied without additional specifications. It is used when a proband is phenotype positive and genotype negative.

##### De novo data (PM6 and PS2)

In SCID patients, de novo variants in AR genes are relatively rare, as with other AR disorders. However, occurrences have been reported.^26^ Additionally, de novo variants in patients with *IL2RG*-associated SCID are more common.^27^ For these cases, the SCID-VCEP specified the PM6 de novo (maternity and paternity not confirmed) and PS2 de novo (maternity and paternity confirmed) criteria following SVI recommendations (Supplemental Table 2).^18^

The SVI-recommended approach bases the strength level for PM6/PS2 on confirmed versus assumed maternity/paternity status, the number of de novo probands, and phenotypic consistency. To achieve the highest level of phenotypic consistency (“phenotype highly specific for gene”), the patient must meet at least the PP4_Moderate criteria. For “phenotype consistent with the gene but not highly specific,” the proband must meet PP4. For “phenotype consistent with gene but not highly specific and high genetic heterogeneity,” the proband must exhibit a SCID phenotype without meeting PP4 criteria.

##### Variant phasing (PM3)

For the utilization of PM3 (detected in trans to a pathogenic variant), the SCID-VCEP followed SVI guidance for points per proband.^18^ This guidance assigns greater point values to probands carrying a pathogenic or likely pathogenic variant confirmed in trans by either parental testing or cloning assays. In contrast, suspected in trans or homozygous occurrences are assigned lower point values (Supplemental Table 3). Additionally, the application of PM3 requires that both variants (or the homozygous variant) in the proband occur at sufficiently rare allele frequencies that they meet the PM2 threshold. The applicability of PM3 to suspected founder variants with allele frequencies exceeding the PM2 threshold is evaluated on a case-by-case basis by the VCEP.

##### Phenotype (PP4)

Recently, PIDTC published updated guidelines for SCID diagnostic criteria, which we deployed for PP4 (patient’s phenotype or family history is highly specific for a disease with a single genetic etiology) assessments.^1^ Because of specificities in the etiology of SCID, according to the gene/pathway affected, in Table 4,^1^ we present the details for PP4 according to each gene. The strength of PP4 applicability is based on a point system derived from clinical features observed in reported cases. If the score is less than 1 point, PP4 is not applied at any strength. For scores from 1 to less than 2 points, PP4 is met at the default level (Supporting). For moderate and strong strengths, the requirements vary by gene: For *ADA*: 2 to <9 points: PP4_Moderate; ≥9 points: PP4_Strong; *IL2RG*: ≥2 to <7 points: PP4_Moderate; ≥8 points: PP4_Strong; *DCLRE1C*: 2 to <7 points: PP4_Moderate; ≥7 points: PP4_Strong; *IL7R* and *JAK3*: 2 to <6 points: PP4_Moderate; ≥6 points: PP4_Strong; *RAG1* and *RAG2*: ≥2 to <4 points: PP4_Moderate; ≥4 points: PP4_Strong. These gene-specific thresholds were defined based on the number and distribution of clinical features reported in the literature for each gene. Because different genes have distinct clinical presentations and diagnostic considerations, the cutoffs for Moderate and Strong evidence levels were calibrated reflecting these gene-specific characteristics.

###### Exclusion of ACMG/AMP criteria.

The exclusion criteria that are universally not applicable across all genes studied are collectively presented here: PP5, BP6, BP1, BP2, BP3, BP4, BP5, and PP2.

We adopted the ClinGen advisement against use of clinical laboratory classification (PP5, BP6).^28^ Furthermore, BP1 (a missense variant in a gene for which primarily truncating variants are known to cause disease) was determined as inapplicable because many pathogenic variants in the 7 genes are indeed missense. This assessment was also based upon ClinVar, where the median percentage of missense variants in the 7 genes was approximately 80%. Additionally, BP2 (observed in trans with a pathogenic variant for a fully penetrant dominant gene/disorder or observed in cis with a pathogenic variant in any inheritance pattern) was defined as inapplicable. The VCEP felt that possibilities might occur wherein the variant of interest is truly pathogenic but by chance is located in cis with a known pathogenic variant along with an unclassified or missing variant on the opposite allele. Because the frequency or likelihood of such an event is not known, the panel elected to curtail use of BP2.

BP3 (in-frame deletions/insertions in a repetitive region without known function) and BP4 (multiple lines of computational evidence suggest no impact on gene or gene product) were also deemed not applicable because none of our genes have known repetitive regions and in silico prediction data and thresholds have not been validated for the genes.

We also removed BP5 (alternative mechanism for disease), given that in very rare circumstances, a patient can carry defects in 2 different genes causing SCID.

Finally, the PP2 criterion (missense variant in a gene that has a low rate of benign missense variation and where missense variants are a common mechanism of disease) was also deemed inapplicable. This assertion was based on the low *z*-score values for all genes (*ADA* = 0.46, *DCLRE1C* = 0.05, *IL7R* = −1.18, *JAK3* = 1.67, *IL2RG* = 1.49, *RAG1* = 1.77, and *RAG2* = 0.57) in the gnomAD missense constraint table, which fall below the SVI recommendation (>3.09) for applying this criterion, as specified in the ClinGen VCEP Standard Operating Protocol.^29^

### Performance of the SCID-VCEP ACMG/AMP specifications in variant classification

The modified SCID-VCEP specifications were tested with a pilot set of 90 variants. The variants were selected from ClinVar and from research and private laboratory data. They included variants with previous assertions of benign/likely benign (B/LB), pathogenic/likely pathogenic (P/LP), VUS, and variants with absent or conflicting interpretations. The selection comprised missense, synonymous, frameshift, splice site, and intronic variants to allow for a comprehensive comparison of how our criteria could be utilized. Case segregation and functional evidence were gathered from limited internal data, as well as available published literature.

**Figure 1:**
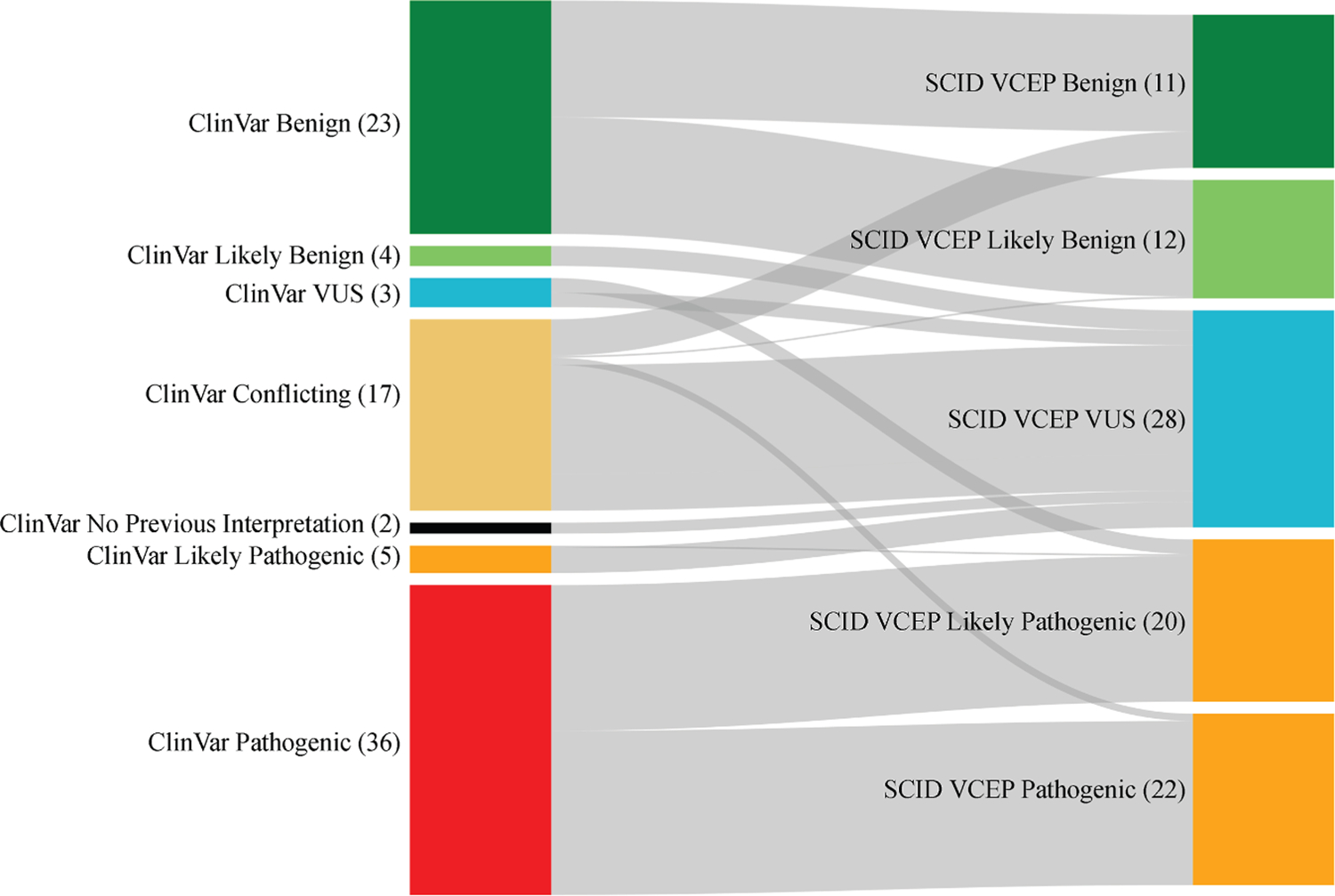
Classification of the 90 selected pilot variants by the original ClinVar assertion (left) and the SCID-VCEP specifics adapted from ACMG/AMP guidelines (right). VUS, variant of uncertain significance. The raw numbers in parentheses represent the total number of variants in each classification category. The variants are approximately equally distributed across the following genes: *ADA* (14), *DCLRE1C* (12), *IL7R* (14), *RAG1* (12), *RAG2* (12), *JAK3* (12), and *IL2RG* (14). ACMG/AMP, American College of Medical Genetics and Genomics/Association for Molecular Pathology; SCID-VCEP, Severe Combined Immunodeficiency Disease Variant Curation Expert Panel.

Utilizing these specifications, 25 variants were classified as pathogenic, 21 as likely pathogenic, 14 as VUS, 18 as likely benign, and 12 as benign. Each of these variants was curated in the ClinGen Variant Curation Interface.^13^ The classifications were approved by the general SCID-VCEP and submitted to the ClinGen Evidence Repository and ClinVar for publication. The changes from previous classifications in ClinVar and the SCID-VCEP classifications can be seen in Figure 1. Supplemental Table 4 contains a complete list of all 90 variants curated, as well as all applicable codes (Supplemental Table 4). After the pilot phase, we initiated a sustained phase, and as of now, 215 variant classifications have been published in ClinVar.

## Discussion

The establishment of the SCID-VCEP represents a significant advancement for the genetic diagnosis of SCID. This initiative is critical for addressing the complex genetic landscape of SCID, in which precise variant classification in genes with established relationships to SCID is pivotal for effective patient care. By providing a robust framework for variant classification, these specifications enhance the accuracy of genetic testing and support more informed clinical decisions.

The application of gene-specific rules for ACMG/AMP variant classification in *ADA*, *DCLRE1C*, *IL2RG*, *IL7R*, *JAK3*, *RAG1*, and *RAG2* has demonstrated encouraging outcomes in a pilot study of 90 variants, with 85% achieving definitive classification (P/LP, B/LB). Regular application of these specifications is likely to decrease the frequency of VUS reported in patients undergoing SCID diagnostic evaluation.

Reclassification of variants can have meaningful clinical consequences. For example, the reclassification of an *ADA* variant from VUS to likely pathogenic can help justify the medical necessity for hematopoietic stem cell transplantation or enzyme replacement or enrollment in an *ADA* gene therapy clinical trial. Additionally, definitive classifications provide improved certainty for genetic counseling, such as recurrence risk assessment, and they support requested authorization for reproductive planning (eg, prenatal or preimplantation genetic testing or in vitro fertilization). Of note, variants reviewed and classified by the SCID-VCEP are assigned an FDA-recognized 3-star review status in ClinVar, which must be respected by third-party payors.

Collectively, these efforts underscore the value of a rigorous, collaborative, and evidence-based approach to variant classification, which can improve the accuracy of SCID diagnosis and support the application of precision medicine.

### Limitations

In evaluating the SCID-VCEP specifications and the variant curation process, it remains essential to recognize the constraints that affect its efficacy and scope. The limitations of this process include the absence of validated thresholds for using in silico tools for these genes. This deficiency constrained our ability to apply PP3 and BP7. Moreover, the lack of broad use of established in vitro functional assays for specific genes, including *IL2RG*, *IL7R*, and *JAK3*, will limit applicability of the functional codes during most classification attempts. Furthermore, the lack of benign variants tested in publications prevents application of PS3 functional evidence at a strong level in the ACMG/AMP evidence framework. These challenges underscore the critical need to develop advanced high-throughput functional assays. Such assays would enable a comprehensive analysis of all potential missense variants in these genes. Where feasible, expedited implementation of functional assays in Clinical Laboratory Improvement Amendments-certified laboratories would allow their results to be used directly in clinical decision making, rather than being restricted to research-only settings. Expanding access to clinically validated functional data could help resolve VUS more efficiently and support improved patient care. Equally important is the need to validate newer computational algorithms designed to assess DNA alterations and their consequential effects on proteins.

### Conclusion

The SCID-VCEP variant classification specifications represent a critical step forward in the accurate interpretation of genetic variants in SCID, offering a gene-specific framework that enhances the precision of clinical diagnoses and supports informed patient care. To support dissemination of these analysis recommendations to commercial testing entities, they are publicly available in CSpec.^12^ Widespread adoption in commercial testing environments, with high volumes of cases and broad ranges of genes analyzed, might make consistent implementation of such gene-specific rules challenging because some rules (eg, for PS3 or PP4) typically require manual review. As such, the SCID-VCEP will continue to classify variants in these genes using these rules and place the curated evidence in ClinVar.

Ongoing refinement and validation of these specifications will ensure that they continue to meet the needs of the clinical community and contribute to better outcomes for patients with SCID. To ensure continued relevance and effectiveness, these specifications should be refined and expanded by incorporating data from underrepresented populations, developing and validating new functional assays, and integrating emerging computational tools. Future directions should focus on fostering collaboration between EPs, clinical laboratories, and researchers to build an adaptable, evidence-based resource that meets the needs of diverse patient populations. By advancing these goals, the SCID-VCEP will contribute significantly to the implementation of precision medicine for patients with SCID and related immunodeficiencies.

## Supplementary Material

Supplementary Table 4

Supplemental Material 1

Supplementary Table 3

Supplementary Table 2

Supplemental Material 2

Supplementary Table 1

Supplemental References

The online version of this article (https://doi.org/10.1016/j.gim.2025.101613) contains supplemental material, which is available to authorized users.

## Figures and Tables

**Table 1:** Population allele frequency thresholds

Genes	Contribution to SCID (%)	BA1 Penetrance: 50% Prevalence = 1/5000^16^ Allelic Heterogeneity = 1	BS1 Penetrance: 100% Prevalence = 1/50,000^14,15^ Allelic Heterogeneity = 1	PM2_Supporting	The Most Frequent Pathogenic Variant^a^
*ADA*	12.8	0.00721	0.00161	0.000174	c.956_960del p.(Glu319Glyfs*3) NC_000020.10: g.43249675_43249679del
*DCLRE1C*	3.2	0.00346	0.00078	0.000032	c.571C>T p.(Arg191*) NC_000010.10:g.14976486G>A
*IL2RG*	30.7	0.01110	0.00249	0.000124	NA
*IL7R*	7.6	0.00566	0.00126	0.000041	c.221+2T>G p.? NC_000005.9:g.35861094T>G
*JAK3*	5.2	0.00447	0.00100	0.000115	c.1351C>T p.(Arg451*) NC_000019.9:g.17950376G>A
*RAG1*	19.2	0.0087	0.0019	0.000102	NA
*RAG2*	19.2	20.00872	50.00195	0.000058	NA

a The most frequently observed pathogenic variant with confirmed pathogenicity in the context of Severe Combined Immunodeficiency (SCID) according to the Variant Classification Expert Panel (VCEP) specifications. The transcripts used are *ADA* (NM_000022.4), *DCLRE1C* (NM_001033855.3), *IL7R* (NM_002185.5), and *JAK3* (NM_000215.4). Genomic positions are based on the GRCh37/hg19 assembly.

**Table 2:** Functional assays (PS3/BS3) approved and strength of evidence

Gene	Rule Code	General Class of Assay	Evidence Level	Readout
*ADA*	BS3	Expressed ADA activity^a^	Supporting	Expressed ADA enzyme activity ≥4.8% of wild-type activity (supports benign interpretation)
	PS3		Supporting	Expressed ADA enzyme activity 0.06%−0.6% of wild-type activity (supports pathogenic interpretation, moderate reduction)
			Moderate	Expressed ADA enzyme activity <0.05% of wild-type activity (supports pathogenic interpretation, severe loss of function)
*DCLRE1C*	PS3	DNA repair activity assay and in vitro V(D)J recombination assay	Supporting	Abnormal result in an in vitro V(D)J recombination assay (defined as <25% of wild-type activity)
			Moderate	Abnormal results in both an in vitro DNA repair activity assay and an in vitro V(D)J recombination assay (defined as <25% of wild-type activity for both assays)
*IL2RG*	PS3	Phosphorylation of JAK3/Co-Immunoprecipitation with JAK3, Cytokine binding, Surface expression of the gamma chain, and Interaction profiling-BioID	Supporting	Abnormal results in at least one approved in vitro assay
*IL7R*	PS3	IL-7-induced JAK3 phosphorylation assay, IL-7 binding assay, and IL-7-induced STAT5 DNA binding/transcriptional induction	Supporting	Abnormal results in at least one approved in vitro assay
*JAK3*	PS3	In vitro kinase assay (JAK3 autophosphorylation)	Supporting	Abnormal result in an in vitro kinase assay (JAK3 autophosphorylation)
*RAG1* and *RAG2*	PS3	In vitro V(D)J recombination assay	Supporting	Abnormal result in an in vitro V(D)J recombination assay, resulting in <25% of wild-type activity
			Moderate	Abnormal result in an in vitro V(D)J recombination assay, resulting in 25%−60% of wild-type activity

PS3 may potentially be applied at the default strength level of strong for evidence from an animal model expressing the variant of interest and recapitulating the SCID phenotype. At least 1 previously observed proband with the variant meeting PP4 is required to apply PS3 at any strength on the basis of a cellular model/in vitro study.

a The same functional assay (ADA enzyme activity) may support either BS3 or PS3, depending on the residual activity observed. Activity ≥ 4.8% of wild-type supports a benign interpretation (BS3). Reduced activity between 0.06% to 0.6% supports pathogenicity at a supporting level (PS3), whereas severely reduced activity (≤0.05%) supports pathogenicity at a moderate level (PS3).

**Table 3:** Critical domains for genes (PM1): Strength of evidence

Gene	Evidence Level	Domains	References
*IL2RG*	Strong	Missense alterations of the following positions: Affecting a conserved cysteine residue: p.Cys62, p.Cys72, p.Cys102, and p.Cys115. Affecting CpG dinucleotides: c.684C (p.Arg224), c.690C (p.Arg226), c.691G (p.Arg691), c.868G (p.Arg285). Affecting the WS×WS motif: p.Trp237, p.Ser238, p.Glu239, p.Trp240, p.Ser241. Affecting a transmembrane domain residue: amino acids 263 to 283, by introducing a charged or polar residue (Asn, Asp, Arg, Cys, His, Glu, Gln, Lys, Ser, Thr, Tyr).	^20^
*JAK3*	Moderate	Missense alterations of 2 pseudo kinase domain (JH2) domain residues: p.Arg651Trp and p.Cys759Arg.	^21^
*RAG1*	Moderate	Missense variant located in: NBD (nonamer-binding domain) domain: amino acids 394 to 460. DDBD (dimerization and DNA-binding domain) domain: amino acids 461 to 517.	^22^ ^22^
	Supporting	Missense variant located elsewhere in: The core domain: amino acids 387 to 1011.	
*RAG2*	Moderate	Missense variant located in: PHD (plant homeodomain) domain: amino acids 414 to 487.	
	Supporting	Missense variant located in: The core domain: amino acazs 1 to 383.	

*ADA* (NM_000022.4), *DCLRE1C* (NM_001033855.3), *IL2RG* (NM_000206.3), *IL7R* (NM_002185.5), *JAK3* (NM_000215.4), *RAG1* (NM_000448.3), and *RAG2* (NM_000536.4).

**Table 4:** Clinical features deemed highly specific for each gene (PP4)

ADA	DCLRE1C	IL2RG	IL7R	JAK3	RAG1	RAG2
Diagnostic criteria for SCID/Leaky SCID/Omenn Syndrome^a^ **0.5 pt**						
SCID gent panel or exome/ genome sequencing conducted^b^ **0.5 pt**	SCID gene panel or exome/ genome sequencing conducted **0.5 pt**	SCID gene panel or exome/ genome sequencing conducted^b^ **1 pt**	SCID gene panel or exome/ genome sequencing conducted^b^ **0.5 pt**	SCID gene panel or exome/ genome sequencing conducted^b^ **0.5 pt**	SCID gene panel or exome/ genome sequencing conducted^b^ **0.5 pt**	SCID gene panel or exome/ genome sequencing conducted^b^ **0.5 pt**
Family history of SCID^c^ **0.5 pt**	Family history of SCID^c^ **0.5 pt**	Family history of SCID^c^ **0.5 pt**	Family history of SCID^c^ **0.5 pt**	Family history of SCID^c^ **0.5 pt**	Family history of SCID^c^ **0.5 pt**	Family history of SCID^c^ **0.5 pt**
Reducet ADA enzyme activity in patient cells (<1% of normal ADA catalytic activity) **1 pt**	Navajo or Apache ancestry **0.25 pt**	XY malt sex **0.5 pt**	Reduced CD127 expression (demonstrated by RT-PCR, Western blot, flow cytometry) **1 pt**	Reduced or constitutive cytokine-induced JAK3 tyrosine phosphorylation in patient cells **1 pt**	Decreased presence of TCRVα7.2 in CD3+ T lymphocytes and/o r mucosa-associated invariant T-cells demonstrated by flow cytometry **0.5 pt**	Decreased presence of TCRVα7.2 in CD3+ T lymphocytes and/or mucosa-associated invariant T-cells demonstrated by flow cytometry **0.5 pt**
Reduced erythrocyte SAHase activity (typically <5% of normal) **1 pt**	Increased cellular radiosensitivity **0.5 pt**	Absent CD132 expression (demonstrated by RT-PCR, Western blot, and flow cytometry) **1 pt**	Reduced IL-7-induced phosphorylation of STAT5 in patient-derived T-cells **1 pt**	Reduced cytokine-induced phosphorylation of STAT5 in patient-derived T or B cells **1 pt**	SCID phenotype corrected by RAG1 gent therapy WITHOUT CNV testing performed **1 pt**	SCID phenotype corrected by RAG2 gent therapy WITHOUT CNV testing performed **1 pt**
SCID phenotype corrected by exogenous ADA supplementation **1 pt**	Decreaset V(D)J recombination **0.5 pt**	Reduced IL2-induced phosphorylation of STAT5 in patient-derived T-cells **1 pt**	Rescue of defective IL-7-receptor-mediated signaling by IL-2 or IL-15 induction **1 pt**	SCID phenotype correctet by JAK3 gene therapy WITHOUT CNV testing performed **1 pt**	SCID phenotype corrected by RAG1 gene therapy WITH CNV testing performed **4 pt**	SCID phenotype corrected by RAG2 gene therapy WITH CNV testing performed **4 pt**
Increased dAdo nucleotides (dATP or dAXP) in pretreatment erythrocytes **2 pt**	Vector-based complementation corrected increased cellular radiosensitivity and/or decreaset V(D)J recombination **2 pt**	Reduced IL21-induced phosphorylation of STAT3 in total lymphocyte or B cells **1 pt**	SCID phenotype corrected by IL7R gene therapy WITHOUT CNV testing performed **1 pt**	SCID phenotype correctet by JAK3 gene therapy WITH CN^d^ testint performed **6 pt**	T^−^B^−^NK^+e^ **0.5 pt**	T^−^B^−^NK^+e^ **0.5 pt**
ADA-SCID phenotype corrected by ADA gene therapy WITHOUT CNV testing performed **1 pt**	SCID phenotype corrected by DCLRE1t gene therapy WITHOUT CNV testing performed **1 pt**	SCID phenotype corrected by IL2RG gene therapy WITHOUT CNV testing performed **1 pt**	SCID phenotype corrected by IL7R gene therapy WITH CNV testing performed **6 pt**	T^−^B+NK^−e^ **0.5 pt**		
ADA-SCID phenotype corrected by ADA gene therapy WITH CNV^d^ testing performed **9 pt**	SCID phenotype corrected by DCLRE1C gene therapy WITH CNV^d^ testing performed **7 pt**	SCID phenotype corrected by IL2RG gene therapy WITH CNV^d^ testing performed **8 pt**	T^−^B^+^NK^+e^ **0.25 pt**			
T^−^B^−^NK^−e^ **0.5 pt**	T^−^B^−^NK^−e^ **0.5 pt**	T^−^B^+^NK^+e^ **0.5 pt**				

a The diagnostic criteria should follow the PIDTC 2022 specification.^1^

b Only applicable if genetic testing did not provide an alternative genetic explanation for SCID/Leaky SCID/Omenn syndrome phenotype.

c Only applicable if SCID gene panel or exome/genome sequencing was conducted on proband and did not provide an alternative genetic explanation for phenotype.

d CNV (Copy number variation) testing is required to consider PP4_Strong in order to certify that the variant in question is the causative for the phenotype and not one CNV event corrected by gene therapy and not identified previously.

e If NK cells are not noted or are present, criteria may still be applied if SCID gene panel or exome/genome sequencing has ruled out alternative causes. If maternal T cells are present, the T lymphocyte profile is still considered to be T- (autologous T cells are absent).

## Data Availability

The ClinGen Severe Combined Immunodeficiency Disease Variant Curation Expert Panel submitted all variants to the ClinVar Database,^30^ including evidence summaries detailing the data used for each classification. The detailed evidence used for the classification of these variants is available in the ClinGen Evidence Repository.^31^ Supplemental Material 3 provides a comprehensive list of variants along with their ClinVar identifiers and the applied codes. Additionally, it includes a curation summary for all variants.
