# Supplementary material for: The ClinGen Severe Combined Immunodeficiency Disease Variant Curation Expert Panel: Specifications for classification of variants in ADA, DCLRE1C, IL2RG, IL7R, JAK3, RAG1, and RAG2
Source: Genet Med. Author manuscript; Available in PMC 2026 May 14. (PMC13175239; doi:10.1016/j.gim.2025.101613)
Supplement: Supplementary Table 3 [file NIHMS2171608-supplement-Supplementary_Table_3.docx]

Supplementary Table 3. Specifications for determining the strength of PM3

| **Classification/Zygosity**  **of other variant ^a^** | **Points per proband** | |
| --- | --- | --- |
|  | **Confirmed in *trans*** | **Phase unknown** |
| Pathogenic or Likely Pathogenic variant | 1.0 | 0.5 (Pathogenic)  0.25 (Likely Pathogenic) |
| Homozygous occurrence  *(max point 1.0)* | 0.5 | N/A |
| Uncertain significance variant  *(max point 0.5)* | 0.25 | 0.0 |
| **Strength of PM3** | **The point total for all probands** | |
| Supporting | 0.5 | |
| Moderate | 1 | |
| Strong | 2 | |
| Very Strong | 4 | |

^a^ All variants should be sufficiently rare (meet PM2 specification)
