# Supplementary material for: The ClinGen Severe Combined Immunodeficiency Disease Variant Curation Expert Panel: Specifications for classification of variants in ADA, DCLRE1C, IL2RG, IL7R, JAK3, RAG1, and RAG2
Source: Genet Med. Author manuscript; Available in PMC 2026 May 14. (PMC13175239; doi:10.1016/j.gim.2025.101613)
Supplement: Supplementary Table 2 [file NIHMS2171608-supplement-Supplementary_Table_2.docx]

Supplementary Table 2. Specifications for determining the strength of PS2/PM6

| **Phenotypic consistency** | **Points per proband** | |
| --- | --- | --- |
|  | ***de novo* with confirmed parental relationships** | ***de novo* with unconfirmed parental relationships** |
| Phenotype highly specific for gene | 2 | 1 |
| Phenotype consistent with gene but not highly specific | 1 | 0.5 |
| Phenotype consistent with gene but not highly specific and highly heterogeneity ^a^ | 0.5 | 0.25 |
| Phenotype not consistent with gene | 0 | 0 |
| **Strength of PS2 or PM6** | **The point total for all probands** | |
| Supporting | 0.5 | |
| Moderate | 1 | |
| Strong | 2 | |
| Very Strong | 4 | |

^a^ Maximum allowable value of 1 may contribute to the overall score
