# Supplementary material for: The ClinGen Severe Combined Immunodeficiency Disease Variant Curation Expert Panel: Specifications for classification of variants in ADA, DCLRE1C, IL2RG, IL7R, JAK3, RAG1, and RAG2
Source: Genet Med. Author manuscript; Available in PMC 2026 May 14. (PMC13175239; doi:10.1016/j.gim.2025.101613)
Supplement: Supplemental References [file NIHMS2171608-supplement-Supplemental_References.docx]

**SUPPLEMENTARY REFERENCES**

1. Akeson AL, Wiginton DA, Dusing MR, States JC, Hutton JJ. Mutant human adenosine deaminase alleles and their expression by transfection into fibroblasts. *J Biol Chem*. 1988;263(31):16291-16296.
2. Arredondo-Vega FX, Santisteban I, Daniels S, Toutain S, Hershfield MS. Adenosine deaminase deficiency: genotype-phenotype correlations based on expressed activity of 29 mutant alleles. *Am J Hum Genet*. 1998;63(4):1049-1059. doi:10.1086/302054
3. Volk T, Pannicke U, Reisli I, et al. DCLRE1C (ARTEMIS) mutations causing phenotypes ranging from atypical severe combined immunodeficiency to mere antibody deficiency. *Hum Mol Genet*. 2015;24(25):7361-7372. doi:10.1093/hmg/ddv437
4. Ege M, Ma Y, Manfras B, et al. Omenn syndrome due to ARTEMIS mutations. *Blood*. 2005;105(11):4179-4186. doi:10.1182/blood-2004-12-4861
5. Felgentreff K, Lee YN, Frugoni F, et al. Functional analysis of naturally occurring DCLRE1C mutations and correlation with the clinical phenotype of ARTEMIS deficiency. *J Allergy Clin Immunol*. 2015;136(1):140-150.e7. doi:10.1016/j.jaci.2015.03.005
6. Pannicke U, Ma Y, Hopfner KP, Niewolik D, Lieber MR, Schwarz K. Functional and biochemical dissection of the structure-specific nuclease ARTEMIS. *EMBO J*. 2004;23(9):1987-1997. doi:10.1038/sj.emboj.7600206
7. Sharfe N, Shahar M, Roifman CM. An interleukin-2 receptor gamma chain mutation with normal thymus morphology. *J Clin Invest*. 1997;100(12):3036-3043. doi:10.1172/JCI119858
8. Kumaki S, Ochs HD, Kuropatwinski KK, et al. A novel mutant gammac chain from a patient with typical phenotype of X-linked severe combined immunodeficiency (SCID) has partial signalling function for mediating IL-2 and IL-4 receptor action. *Clin Exp Immunol*. 1999;115(2):356-361. doi:10.1046/j.1365-2249.1999.00792.x
9. Arcas-García A, Garcia-Prat M, Magallón-Lorenz M, et al. The IL-2RG R328X nonsense mutation allows partial STAT-5 phosphorylation and defines a critical region involved in the leaky-SCID phenotype. *Clin Exp Immunol*. 2020;200(1):61-72. doi:10.1111/cei.13405
10. Kumaki S, Ochs HD, Timour M, et al. Characterization of B-cell lines established from two X-linked severe combined immunodeficiency patients: interleukin-15 binds to the B cells but is not internalized efficiently. *Blood*. 1995;86(4):1428-1436.
11. Tuovinen EA, Grönholm J, Öhman T, et al. Novel Hemizygous IL2RG p.(Pro58Ser) Mutation Impairs IL-2 Receptor Complex Expression on Lymphocytes Causing X-Linked Combined Immunodeficiency. *J Clin Immunol*. 2020;40(3):503-514. doi:10.1007/s10875-020-00745-2
12. Roifman CM, Zhang J, Chitayat D, Sharfe N. A partial deficiency of interleukin-7R alpha is sufficient to abrogate T-cell development and cause severe combined immunodeficiency. *Blood*. 2000;96(8):2803-2807.
13. Puel A, Ziegler SF, Buckley RH, Leonard WJ. Defective IL7R expression in T(-)B(+)NK(+) severe combined immunodeficiency. *Nat Genet*. 1998;20(4):394-397. doi:10.1038/3877
14. Roberts JL, Lengi A, Brown SM, et al. Janus kinase 3 (JAK3) deficiency: clinical, immunologic, and molecular analyses of 10 patients and outcomes of stem cell transplantation. *Blood*. 2004;103(6):2009-2018. doi:10.1182/blood-2003-06-2104
15. Corneo B, Moshous D, Güngör T, et al. Identical mutations in RAG1 or RAG2 genes leading to defective V(D)J recombinase activity can cause either T-B-severe combined immune deficiency or Omenn syndrome. *Blood*. 2001;97(9):2772-2776. doi:10.1182/blood.v97.9.2772
16. Lee YN, Frugoni F, Dobbs K, et al. A systematic analysis of recombination activity and genotype-phenotype correlation in human recombination-activating gene 1 deficiency. J Allergy Clin Immunol. 2014;133(4):1099-1108. doi:10.1016/j.jaci.2013.10.007
17. Couëdel C, Roman C, Jones A, Vezzoni P, Villa A, Cortes P. Analysis of mutations from SCID and Omenn syndrome patients reveals the central role of the Rag2 PHD domain in regulating V(D)J recombination. *J Clin Invest*. 2010;120(4):1337-1344. doi:10.1172/JCI41305
18. Tirosh I, Yamazaki Y, Frugoni F, et al. Recombination activity of human recombination-activating gene 2 (RAG2) mutations and correlation with clinical phenotype. *J Allergy Clin Immunol*. 2019;143(2):726-735. doi:10.1016/j.jaci.2018.04.027
19. Dvorak CC, Haddad E, Heimall J, et al. The diagnosis of severe combined immunodeficiency (SCID): The Primary Immune Deficiency Treatment Consortium (PIDTC) 2022 Definitions. *J Allergy Clin Immunol*. 2023;151(2):539-546. doi:10.1016/j.jaci.2022.10.022
